# Balloon-Expandable Transcatheter Aortic Valve Implantation for LVAD-Associated Native Aortic Insufficiency: A Single-Center Experience

**DOI:** 10.3390/jcdd13080355

**Published:** 2026-07-29

**Authors:** Bilge Duran Karaduman, Telat Keleş, Özlem Özcan Çelebi, Sinan Sabit Kocabeyoğlu, Abdulkadir Yılmaz, Mustafa Akdi, Ümit Kervan

**Affiliations:** 1 Department of Cardiology, Ankara Bilkent City Hospital, 06800 Ankara, Türkiye; drtelatkeles@yahoo.com (T.K.); ozlemozcan.celebi@sbu.edu.tr (Ö.Ö.Ç.); 2 Department of Cardiovascular Surgery, Ankara Bilkent City Hospital, 06800 Ankara, Türkiye; sinansabit.kocabeyoglu@sbu.edu.tr (S.S.K.); abdulkadir.yilmaz@saglik.gov.tr (A.Y.); mustafa.akdi@saglik.gov.tr (M.A.); umit.kervan@sbu.edu.tr (Ü.K.)

**Keywords:** aortic insufficiency, left ventricular assist device, transcatheter aortic valve implantation, balloon-expandable valve, anatomy-driven strategy

## Abstract

Background: Aortic insufficiency (AI) during long-term left ventricular assist device (LVAD) support creates a maladaptive recirculatory loop that impairs forward flow, increases left ventricular filling pressures, leading to progressive congestion, and accelerates right ventricular dysfunction. Surgical correction carries high risk, making transcatheter aortic valve implantation (TAVI) an alternative strategy, although outcomes are challenged by complex annular geometry and the absence of calcific anchoring. Methods: We performed a retrospective single-center analysis of consecutive LVAD-supported patients undergoing TAVI for native AI. Annular sizing was area-based with a predefined oversizing strategy of approximately 15–30%, adjusted to annular geometry. Balloon-expandable valves were implanted using controlled deployment under rapid pacing and temporary LVAD flow reduction. Outcomes were assessed according to VARC-3 definitions. Results: Fourteen patients underwent TAVI. The cohort demonstrated high anatomical complexity, including annular eccentricity (median 0.21 [IQR 0.19–0.23]), horizontal aorta (42.9%), and flared left ventricular outflow tract (LVOT) morphology (50%). Median annular area was 528 mm^2^ (IQR 487–574), with area-derived diameter 25.9 mm (IQR 24.9–27.0). Mean oversizing was 21.9% (median 20.0%; range 15.1–30.3%). Technical and VARC-3 device success were achieved in 71.4%. Second valve implantation occurred in 28.6%. Procedural mortality was 0%. In-hospital mortality (21.4%) was related to low cardiac output and multi-organ dysfunction rather than device failure. No survivor had moderate or greater residual AI at discharge. Conclusions: In anatomically complex LVAD patients, an anatomy-driven balloon-expandable TAVI strategy achieved elimination of clinically significant AI with zero procedural mortality. In our interpretation, mortality reflected the advanced stage of heart failure at the time of intervention rather than acute device-related failure.

## 1. Introduction

Left ventricular assist devices (LVADs) have become an established therapy for patients with advanced heart failure, serving both as bridge to transplantation and destination therapy. Registry data show that aortic insufficiency (AI) develops in approximately 30% of patients within 2–3 years after LVAD implantation [1,2]. In continuous-flow LVAD support, AI may create a recirculatory loop that reduces effective forward flow and increases left ventricular filling pressures, leading to progressive congestion [3].

Surgical correction of AI remains a definitive therapeutic option; however, redo cardiac surgery in LVAD recipients is associated with substantial morbidity and mortality, including increased risks of bleeding, right ventricular failure, and impaired wound healing [4,5]. Consequently, transcatheter aortic valve implantation (TAVI) has emerged as a less invasive alternative for selected patients with symptomatic LVAD-associated AI who are deemed unsuitable for repeat surgery [6].

Nevertheless, TAVI in LVAD-associated AI remains technically challenging. In contrast to calcific aortic stenosis, AI in this population is frequently characterized by minimal or absent annular and leaflet calcification, annular dilatation, and remodeling of the left ventricular outflow tract (LVOT), limiting intrinsic valve anchoring and increasing susceptibility to valve malposition, migration, and residual regurgitation [7]. Published LVAD–TAVI series remain limited and heterogeneous in valve platform and procedural strategy [8,9,10,11,12]. Device-specific data for balloon-expandable valves remain scarce.

The present study reports a retrospective single-center experience of TAVI for native AI in LVAD-supported patients, focusing on anatomical determinants, procedural feasibility, and early clinical outcomes using a homogeneous balloon-expandable strategy.

## 2. Methods

### 2.1. Study Design, Ethics, and Patient Population

This retrospective single-center study was conducted at a tertiary referral center with expertise in advanced heart failure, mechanical circulatory support, and structural heart interventions. Consecutive patients supported with durable continuous-flow LVADs who underwent TAVI for the treatment of native AI were included.

During the study period, 172 patients underwent HeartMate 3 (HM3 Abbott Laboratories, Abbott Park, IL, USA) LVAD implantation at our institution. In total, 242 LVAD-supported patients were followed, including patients implanted at our center as well as those implanted previously and referred for longitudinal follow-up. Among this overall LVAD population, aortic insufficiency of any grade developed in approximately 36% of patients, while moderate-to-severe or severe AI occurred in approximately 16%. The present study includes the subset of patients with clinically significant native AI who underwent TAVI following multidisciplinary heart team evaluation.

All patients were hospitalized and evaluated at our institution and were referred for TAVI assessment by an experienced cardiovascular surgery team with dedicated expertise in transplantation and LVAD implantation, as part of a multidisciplinary heart team approach.

The study protocol was approved by the Institutional Ethics Committee (Decision No: TABED 2-26-1919), and written informed consent was waived due to the retrospective design. The study was conducted in accordance with the Declaration of Helsinki, and all patient data were anonymized prior to analysis.

Clinically significant AI was defined as moderate-to-severe or severe AI associated with clinical deterioration considered predominantly attributable to AI after exclusion of alternative causes, based on comprehensive clinical, echocardiographic, and hemodynamic assessment. The decision to perform TAVI was made by a multidisciplinary heart team after integration of all available clinical and imaging findings.

Patients were considered candidates for TAVI if they had clinically significant native aortic insufficiency associated with heart failure symptoms or hemodynamic deterioration, were deemed to be at high or prohibitive surgical risk for repeat surgery, and had anatomical characteristics considered suitable for transcatheter treatment based on multimodality imaging. During the study period, three patients with clinically significant LVAD-associated aortic insufficiency underwent surgical correction. The present study therefore includes only patients who were selected for transcatheter treatment following multidisciplinary heart team evaluation.

Patients were eligible if they had:

1. Durable continuous-flow LVAD support;
2. Echocardiographically documented clinically significant native AI;
3. Underwent TAVI following multidisciplinary heart team evaluation.

Patients undergoing other aortic valve interventions were excluded.

### 2.2. Baseline Clinical and Imaging Assessment

Baseline clinical data were obtained from electronic records. All patients underwent comprehensive transthoracic and/or transesophageal echocardiographic assessment before the procedure. AI severity was assessed using a literature-based multiparametric echocardiographic approach adapted to continuous-flow LVAD physiology. Parameters considered included color Doppler jet characteristics, vena contracta width, jet width-to-LVOT ratio, pressure half-time, diastolic flow reversal in the descending aorta, aortic valve opening frequency, and the duration of the regurgitant jet throughout the cardiac cycle. When technically available, LVAD outflow graft Doppler parameters, including the systolic-to-diastolic velocity ratio and diastolic flow acceleration, were also considered. Echocardiographic findings were interpreted together with the patients’ clinical presentation and hemodynamic status to determine the clinical significance of AI. In selected cases, LVAD speed was temporarily reduced to avoid underestimation of AI severity [13]. Multidetector computed tomography (MDCT) was performed for anatomical assessment when feasible; in patients with renal dysfunction, annular sizing was performed using three-dimensional transesophageal echocardiography (3D-TEE).

3D-TEE measurements of the annulus, left ventricular outflow tract (LVOT), sinus of Valsalva, and sino-tubular junction were obtained, and annular diameter was derived from annular area measurements. Oversizing (%) was calculated as (nominal valve area/annular area − 1) × 100.

### 2.3. Heart Team Evaluation and Procedural Planning

All cases were reviewed by a multidisciplinary heart team. Valve sizing was guided by annular area and perimeter measurements, landing zone morphology, and operator experience. Conventional sizing algorithms used for calcific aortic stenosis were not directly applied.

A predefined target oversizing strategy of approximately 15–30% relative to annular area was applied, individualized according to landing zone compliance and annular geometry.

LVOT morphology was categorized using diameter-based measurements. LVOT morphology was defined as tubular (≤5% difference), flared (>5% larger), or tapered (>5% smaller) relative to annular diameter.

### 2.4. Transcatheter Aortic Valve Implantation Procedure

All procedures were performed in the cardiac catheterization laboratory under fluoroscopic and TEE guidance. Balloon-expandable transcatheter heart valves (Myval™ Transcatheter Heart Valve, Meril Life Sciences Pvt. Ltd., Vapi, India) were implanted in all patients.

During deployment, LVAD flow was reduced and rapid ventricular pacing applied to facilitate controlled positioning and reduce migration risk, consistent with prior LVAD–TAVI strategies [6,10].

Controlled, stepwise balloon inflation under rapid pacing was favored to allow fine positional adjustment in the absence of calcific anchoring.

Valve stability and residual AI were assessed using fluoroscopy and TEE before post-dilatation. Post-dilatation was performed only when necessary. In cases of valve instability or significant residual AI, prompt implantation of a second valve was undertaken. The need for a second valve was recorded as a marker of procedural complexity.

### 2.5. Postprocedural Assessment and Follow-Up

Clinical and echocardiographic evaluation prior to discharge assessed residual AI, ventricular function, and complications.

Follow-up was performed at 1, 3, and 6 months and continued thereafter. Outcomes included all-cause mortality, heart failure-related hospitalization, and clinical status.

### 2.6. Statistical Analysis

Primary descriptive endpoints included technical success, device success according to VARC-3 criteria [14], need for second valve implantation, and 30-day mortality. Continuous variables are presented as mean ± SD or median (IQR), according to distribution. Categorical variables are expressed as counts and percentages. Normality of continuous variables was assessed using visual inspection and the Shapiro–Wilk test. Statistical analyses were performed using SPSS version 24.0 (IBM Corp., Armonk, NY, USA).

Given the descriptive nature and limited sample size, no formal comparative testing was performed.

## 3. Results

During the study period, 39 patients developed moderate-to-severe or severe LVAD-associated aortic insufficiency. Of these, 14 underwent TAVI, three underwent surgical treatment, and 22 were managed conservatively with medical therapy. Among the medically managed patients, the median survival after the diagnosis of clinically significant aortic insufficiency was 3.0 months (IQR, 2.0–5.75 months).

Fourteen LVAD-supported patients underwent TAVI for native AI. Mean age was 52.5 ± 15.1 years, and 92.9% were male. Heart failure etiology was ischemic in 42.9%, nonischemic in 50.0%, and postpartum in 7.1%. All patients were supported with HeartMate 3 (HM3) devices. LVAD indication was bridge-to-transplant in 42.9% and destination therapy in 57.1%. The median interval from LVAD implantation to TAVI was 32.5 months (IQR 29.8–47.8). NYHA class III–IV symptoms were present in 71.4% of patients, and 14.3% underwent urgent intervention due to cardiogenic shock. Mean eGFR was 52 ± 15.8 mL/min/1.73 m^2^ (Table 1).

Median aortic annular area was 528 mm^2^ (IQR 487–574), annular perimeter was 82.5 mm (IQR 79.1–85.6), and area-derived annular diameter was 25.9 mm (IQR 24.9–27.0). Minimal calcification was present in 28.6%, while 71.4% had no detectable calcification. LVOT morphology was evenly distributed between flared and tubular configurations (50% each). The annular eccentricity index was 0.21 (IQR 0.19–0.23). The median horizontal aorta angle was 48° (IQR 43–55.5), with 42.9% demonstrating a horizontal configuration (≥50°) (Table 2).

All procedures were performed transfemorally under TEE guidance. Balloon-expandable Myval™ transcatheter heart valves were implanted in all patients (24.5 mm in 14.3%, 27.5 mm in 28.6%, 29 mm in 42.9%, and 32 mm in 14.3%). Sizing was area-based with a median oversizing of 20.0% (mean 21.9%; range 15.1–30.3%). Rapid pacing and temporary LVAD flow reduction were applied in all procedures. Post-dilatation was required in 28.6%. Second valve implantation was necessary in four patients (28.6%), due to migration in two and significant PVL in two cases. All patients requiring a second valve had flared LVOT morphology (4/7, 57.1%), whereas no second valve was required in tubular LVOT configuration (0/7). Technical success was achieved in 10 patients (71.4%), and VARC-3 device success was achieved in 10 patients (71.4%) (Table 3).

At the end of the procedure, no patient had moderate or greater residual AI. Residual AI was none in 71.4%, trace in 21.4%, and mild in 7.1%. Among the 11 patients discharged alive, no patient had moderate or greater AI; 90.9% had no residual AI and 9.1% had trace AI. No in-hospital stroke or TIA occurred. One patient (7.1%) experienced a major vascular complication requiring surgical intervention. No major bleeding, new permanent pacemaker implantation, or LVAD pump thrombosis occurred. Right heart failure requiring inotropic support occurred in 21.4% (Table 4).

In-hospital mortality occurred in three patients (21.4%). Procedural mortality was 0%, as all patients left the catheterization laboratory hemodynamically stable. One patient experienced valve embolization requiring surgical intervention and subsequently died during the same hospitalization. The remaining two deaths were attributed to refractory low cardiac output and progressive multi-organ dysfunction. Thirty-day mortality was 21.4% (Table 4).

Median follow-up duration was 9.0 months (IQR 1.8–16.0; range 0.26–60.0 months). No recurrent hospitalization occurred within 30 days among discharged patients. During the 3–6 months follow-up (*n* = 11), hospitalization for low cardiac output occurred in 27.3%, and driveline infection in 9.1% (Table 4).

## 4. Discussion

In this single-center series of LVAD-supported patients undergoing transcatheter treatment for native AI, we demonstrate that a standardized area-based oversizing strategy combined with controlled balloon-expandable deployment achieved consistent elimination of moderate or greater residual AI at discharge among survivors. Procedural complexity remained substantial, with second valve implantation in 28.6% and VARC-3 device success of 71.4%. All patients requiring valve-in-valve therapy shared minimal annular calcification and flared LVOT anatomy, highlighting the role of anatomical substrate in procedural stability.

### 4.1. LVAD-Associated AI: Hemodynamic Impact and Timing of Intervention

In continuous-flow LVAD recipients, AI creates a recirculatory loop that impairs systemic perfusion [15]. In our cohort, most patients presented with advanced NYHA III–IV symptoms, suggesting delayed correction may increase vulnerability. Earlier referral before progression to advanced NYHA IV status and overt right ventricular failure may therefore be critical in optimizing outcomes.

### 4.2. Echocardiographic Assessment in the LVAD Setting

Assessment of AI severity in LVAD-supported patients is frequently underestimated by conventional echocardiographic criteria due to altered loading conditions and continuous-flow physiology. Grinstein et al. demonstrated that reliance on color Doppler alone may underestimate regurgitant burden and advocated a multiparametric approach incorporating vena contracta, pressure half-time, and diastolic flow reversal [13]. In our series, systematic use of TEE guidance and multimodality imaging was central to procedural planning and post-implantation assessment.

### 4.3. Surgical Risk and the Role of Transcatheter Therapy

Redo surgical correction carries substantial perioperative risk in LVAD-supported patients [16,17]. Comparative analyses suggest that transcatheter approaches may offer lower perioperative morbidity with comparable survival [18].

### 4.4. Second Valve Requirement and Anatomical Determinants

Second valve implantation occurred in 28.6% of patients, consistent with prior LVAD–TAVI series [10,11]. Absence of annular calcification increases procedural complexity and need for additional valve implantation [19,20]. In our cohort, flared LVOT morphology was consistently associated with second valve requirement, reinforcing the importance of landing zone geometry [8]. In our cohort, all patients requiring a second valve shared two important anatomical features: flared LVOT morphology and minimal or absent annular calcification. Together, these characteristics created a less constrained landing zone with reduced anchoring support despite intentional oversizing, predisposing to valve migration or clinically significant residual paravalvular regurgitation. These findings suggest that landing zone geometry, rather than oversizing alone, may be a major determinant of procedural stability in LVAD-associated native AI.

One patient experienced valve embolization into the left ventricle in the setting of flared LVOT anatomy and absent calcification, reinforcing that patient-specific geometry—rather than valve platform alone—may be the dominant determinant of anchoring success.

### 4.5. Balloon-Expandable Versus Self-Expanding Platforms

The optimal valve platform for LVAD-associated AI remains debated. Contemporary mixed-platform series have not consistently demonstrated clear superiority of one platform over another in this setting [10].

Balloon-expandable valves may provide advantages including predictable radial force, precise implantation depth control under rapid pacing, and immediate full expansion that may reduce delayed migration risk. In our experience, slow and controlled balloon deployment with temporary LVAD flow reduction was critical to minimizing displacement.

Dedicated devices for aortic regurgitation may improve anchoring in non-calcified anatomies. Dedicated devices such as the J-valve have been explored in LVAD-associated native aortic regurgitation. Garcia et al. reported the feasibility of J-valve implantation in LVAD-associated AR [21]. The 27-patient series by Hinkov et al. suggested improved device success when AI-dedicated platforms or landing zone preparation strategies were employed [11].

### 4.6. Mortality in Context of Contemporary Literature

In-hospital mortality in our cohort (21.4%) aligns with reported ranges in LVAD–TAVI series, particularly among patients presenting with advanced heart failure [7]. Contemporary reports describe early mortality between approximately 11% and 29%, with second valve implantation rates between 19% and 36% [10,11]. Recently, Bashir et al. reported similar findings in a contemporary institutional series, demonstrating high procedural feasibility but persistent anatomical challenges and early mortality driven primarily by advanced heart failure rather than device failure [22]. Importantly, procedural mortality was 0%, as all patients left the catheterization laboratory hemodynamically stable. Subsequent deaths were driven by refractory low cardiac output and multi-organ dysfunction rather than acute device-related failure. Thirty-day mortality was likewise 21.4% (three patients). No additional mortality occurred among surviving patients during 3- and 6-month follow-up. These findings suggest that the observed in-hospital mortality primarily reflected the advanced clinical condition of the treated patients rather than procedural complications. All patients survived the procedure itself, and the subsequent deaths occurred secondary to refractory low cardiac output and progressive multi-organ dysfunction despite successful valve implantation. The absence of procedural mortality and the lack of additional deaths after hospital discharge further support the interpretation that early mortality was predominantly related to the severity of the underlying heart failure rather than failure of the transcatheter procedure.

This cohort demonstrated high-risk anatomical characteristics: median annular eccentricity index was 0.21 (IQR 0.19–0.23), horizontal aorta configuration (≥50°) was present in 42.9%, and 50% demonstrated flared LVOT morphology. These characteristics reflect a challenging landing zone with limited intrinsic anchoring support. Despite this high-risk substrate, complete elimination of clinically significant AI was achieved among survivors.

A pooled analysis of 358 patients comparing TAVI and SAVR in LVAD-associated AI reported lower in-hospital composite complications and reduced bleeding with transcatheter therapy, with comparable overall survival [23]. These data further support consideration of a transcatheter-first approach in appropriately selected LVAD-supported patients.

### 4.7. Pacemaker Requirement

Pacemaker implantation rates after TAVI for pure native aortic regurgitation have been reported to range between approximately 7.5% and 27.3%, depending on device type and conduction disturbance predictors [24]. In our series, no patient required new permanent pacemaker implantation, which may reflect controlled oversizing and careful implantation depth.

### 4.8. Limitations

This study is limited by its retrospective design and modest sample size. Nonetheless, it represents a contemporary homogeneous balloon-expandable LVAD–TAVI series with standardized technique and anatomy-driven planning. Larger multicenter prospective studies are needed to define optimal device selection, timing of intervention, and standardized sizing strategies in LVAD-associated AI.

## 5. Conclusions

In this anatomically complex and clinically advanced LVAD-supported population, TAVR using a standardized, anatomy-driven balloon-expandable strategy achieved complete elimination of clinically significant AI among surviving patients with zero procedural mortality. Early mortality reflected the advanced disease stage rather than acute device failure. Careful oversizing, controlled deployment, and multidisciplinary heart team assessment appear to be important components of a successful treatment strategy in this high-risk population.

## Figures and Tables

**Table 1:** Baseline patient characteristics.

Variable	Overall (*n* = 14)
Age, years	52.5 ± 15.1
Female sex, *n* (%)	1 (7.1%)
Heart failure etiology (ICM/NICM/other), *n* (%)	ICM: 6 (42.9%) Postpartum: 1 (7.1%) NICM: 7 (50.0%)
LVAD type (HM3/HMII/HVAD/other), *n* (%)	100% HM3
LVAD indication (BTT/DT), *n* (%)	6 (42.9%) BTT 8 (57.1%) DT
Time from LVAD implantation to TAVI, months	32.5 (IQR 29.8–47.8)
NYHA class (pre-TAVI), *n* (%)	II: 4 (28.6%) III: 6 (42.9%) IV: 4 (28.6%)
Cardiogenic shock/in-patient urgent TAVI, *n* (%)	2 (14.3%)
Baseline eGFR, mL/min/1.73 m^2^ Baseline AKI, *n* (%)	52 ± 15.8 4 (28.6%)
History of GI bleeding, *n* (%)	0 (0%)
Baseline MR severity, *n* (%)	None: 4 (28.6%) Mild: 1 (7.1%) Moderate: 2 (14.3%) Moderate-to-severe: 3 (21.4%) Severe: 4 (28.6%)
Baseline TR severity, *n* (%)	None: 4 (28.6%) Mild: 1 (7.1%) Moderate: 2 (14.3%) Moderate-to-severe: 3 (21.4%) Severe: 4 (28.6%)
Baseline RV dysfunction (TAPSE/RVFAC)	TAPSE: 14.7 ± 3.3 mm RVFAC: 27.7 ± 6.9% RVTDI S’: 7.4 ± 1.3

Abbreviations: LVAD = Left ventricular assist device; TAVI = Transcatheter aortic valve implantation; ICM = Ischemic cardiomyopathy; NICM = Non-ischemic cardiomyopathy; BTT = Bridge to transplant; DT = Destination therapy; NYHA = New York Heart Association; eGFR = Estimated glomerular filtration rate; AKI: Acute kidney injury; GI = Gastrointestinal; MR = Mitral regurgitation; TR = Tricuspid regurgitation; RV = Right ventricular; TAPSE = Tricuspid annular plane systolic excursion; RVFAC = Right ventricular fractional area change; and RVTDIs = Right ventricular tissue Doppler systolic velocity (S′).

**Table 2:** Baseline imaging and anatomic characteristics.

Variable	Overall (*n* = 14)
Preprocedural imaging used for sizing	3D-TEE: 4 (28.6%) CT: 10 (71.4%)
Aortic valve/annular calcification	None: 10 (71.4%) Minimal: 4 (28.6%) Mild: 0 (0%) Moderate: 0 (0%)
Annular area, mm^2^	Median: 528 (IQR 487–574)
Annular perimeter, mm	Median: 82.5 (IQR 79.1–85.6)
Annular diameter (area-derived), mm	Median: 25.9 (IQR 24.9–27.0)
LVOT morphology, *n* (%)	Flared: 7 (50.0%) Tubular: 7 (50.0%) Tapered: 0 (0%)
Median annulus eccentricity index (perimeter),	0.21 (IQR 0.19–0.23)
Horizontal aorta (angle)	48° (IQR 43–55.5) ≥50°: 6 (42.9%)
Pre-TAVI AI severity	Moderate-to-severe: 6 (42.9%) Severe: 8 (57.1%)

Abbreviations: 3D-TEE = Three-dimensional transesophageal echocardiography; CT = Computed tomography; LVOT = Left ventricular outflow tract; IQR = Interquartile range; and AI = Aortic insufficiency.

**Table 3:** Procedural details and strategy.

Variable	Overall (*n* = 14)
Access route	Transfemoral: 14 (100%) Other: 0 (0%)
Anesthesia	General: 14 (100%) Deep sedation: 0 (0%)
Imaging guidance	TEE: 14 (100%)
Valve platform (Myval) size distribution	24.5 mm: 2 (14.3%) 27.5 mm: 4 (28.6%) 29 mm: 6 (42.9%) 32 mm: 2 (14.3%)
Sizing modality	MDCT: 10 (71.4%) 3D-TEE: 4 (28.6%)
Oversizing (%)	Mean: 21.9% Median: 20.0% Range: 15.1–30.3%
Rapid pacing used	14 (100%)
LVAD flow reduction during deployment	14 (100%)
Post-dilatation performed	4 (28.6%)
Second valve required	4 (28.6%)
Reason for second valve	Migration: 2 (14.3%) Significant PVL: 2 (14.3%)

Abbreviations: TEE = Transesophageal echocardiography; MDCT = Multidetector computed tomography; PVL = Paravalvular leak; and LVAD = Left ventricular assist device.

**Table 4:** Procedural outcomes (VARC-3).

Outcome	Overall (*n* = 14)
Technical success	10 (71.4%)
Device success (VARC-3)	10 (71.4%)
Residual AI at end of case	None: 10 (71.4%) Trace: 3 (21.4%) Mild: 1 (7.1%) Moderate+: 0 (0%)
Residual AI at discharge (*n* = 11)	None: 10 (90.9%) Trace: 1 (9.1%)
Stroke/TIA (in-hospital)	0 (0%)
Major vascular complication	1 (7.1%)
Major bleeding	0 (0%)
Acute kidney injury (post-procedural)	0 (0%)
New permanent pacemaker	0 (0%)
Right heart failure requiring inotropes	3 (21.4%)
LVAD pump thrombosis	0 (0%)
Procedural mortality	0 (0%)
In-hospital mortality	3 (21.4%)
30-day mortality	3 (21.4%)
HF hospitalization within 30 days (*n* = 11)	0 (0%)
Recurrent hospitalization (3–6 months, *n* = 11)	Low cardiac output: 3 (27.3%) Driveline infection: 1 (9.1%)
Median follow-up duration, months:	9.0 (IQR 1.8–16.0)

Abbreviations: AI = Aortic insufficiency; TIA = Transient ischemic attack; VARC-3 = Valve Academic Research Consortium-3; and HF = Heart failure.

## Data Availability

The data presented in this study are available from the corresponding author upon reasonable request. The data are not publicly available due to institutional privacy and ethical restrictions.
